# A Single Dose Oral Azithromycin versus Intramuscular Benzathine Penicillin for the Treatment of Yaws-A Randomized Non Inferiority Trial in Ghana

**DOI:** 10.1371/journal.pntd.0005154

**Published:** 2017-01-10

**Authors:** Cynthia Kwakye-Maclean, Nsiire Agana, John Gyapong, Priscilia Nortey, Yaw Adu-Sarkodie, Esther Aryee, Kingsley Asiedu, Roland Ballard, Fred Binka

**Affiliations:** 1 Ga West Municipal Health Administrations, Ghana Health Service, Amasaman, Ghana; 2 National Yaws Eradication Program, Ghana Health Service, Accra, Ghana; 3 Department of Epidemiology, School of Public Health, University of Ghana, Legon, Ghana; 4 Kwame Nkrumah University of Science and Technology, Kumasi, Ghana; 5 National Public Health Reference Laboratory, Korle-Bu Teaching Hospital, Accra Ghana; 6 Global Yaws Eradication Programme, Department of Control of Neglected Tropical Diseases, World Health Organization, Geneva, Switzerland; 7 Center for Global Health, Centers for Disease Control and Prevention, Atlanta, Georgia, United States of America; Fondation Raoul Follereau, FRANCE

## Abstract

**Background:**

Yaws is a treponemal infection that was almost eradicated fifty years ago; however, the disease has re-emerged in a number of countries including Ghana. A single-dose of intramuscular benzathine penicillin has been the mainstay of treatment for yaws. However, intramuscular injections are painful and pose safety and logistical constraints in the poor areas where yaws occurs. A single center randomized control trial (RCT) carried out in Papua New Guinea in 2012 demonstrated the efficacy of a single-dose of oral azithromycin for the treatment of yaws. In this study, we also compared the efficacy of a single oral dose of azithromycin as an alternative to intramuscular benzathine penicillin for the treatment of the disease in another geographic setting.

**Methodology:**

We conducted an open-label, randomized non-inferiority trial in three neighboring yaws-endemic districts in Southern Ghana. Children aged 1–15 years with yaws lesions were assigned to receive either 30mg/kg of oral azithromycin or 50,000 units/kg of intramuscular benzathine penicillin. The primary end point was clinical cure rate, defined as a complete or partial resolution of lesions 3 weeks after treatment. The secondary endpoint was serological cure, defined as at least a 4-fold decline in baseline RPR titre 6 months after treatment. Non- inferiority of azithromycin treatment was determined if the upper bound limit of a 2 sided 95% CI was less than 10%.

**Findings:**

The mean age of participants was 9.5 years (S.D.3.1, range: 1–15 years), 247(70%) were males. The clinical cure rates were 98.2% (95% CI: 96.2–100) in the azithromycin group and 96.9% (95% CI: 94.1–99.6) in the benzathine penicillin group. The serological cure rates at 6 months were 57.4% (95% CI: 49.9–64.9) in the azithromycin group and 49.1% (95% CI: 41.2–56.9) in the benzathine penicillin group, thus achieving the specified criteria for non-inferiority.

**Conclusions:**

A single oral dose of azithromycin, at a dosage of 30mg/kg, was non-inferior to a single dose of intramuscular benzathine penicillin for the treatment of early yaws among Ghanaian patients, and provides additional support for the WHO policy for use of oral azithromycin for the eradication of yaws in resource-poor settings.

**Trial Registration:**

Pan African Clinical Trials Registry PACTR2013030005181 http://www.pactr.org/

## Author Summary

Yaws is a tropical infection caused by a bacterium closely related to that which causes syphilis. It is transmitted from person to person through skin to skin contact and often causes papillomatous and ulcerative skin lesions, usually in young children. Without treatment, it can lead to deformities and disabilities. In the past, treatment of cases and their contacts and mass treatment of whole communities has been conducted using single doses of long acting penicillin. This treatment is inexpensive and does not pose a problem with adherence. However, the injections are painful and make it difficult to gain the cooperation of children. In addition, it requires trained health workers to safely administer treatment in poor-resource settings where yaws commonly occurs. In this study one group of children aged 1–15 years with clinically and serologically confirmed yaws received the standard treatment of a single injection of benzathine penicillin. A second group of children were treated with a single dose of oral azithromycin. The children were followed up at 3 weeks to assess healing of lesions, and at 3 and 6 months respectively to monitor serological indicators of infection. Our conclusion is that single dose oral azithromycin is as effective as a single injection of benzathine penicillin for the treatment of early yaws in Ghana and confirms the findings of a previous study undertaken in Papua New Guinea.

## Introduction

### Background

Yaws is a relapsing non-venereal treponematosis caused by *Treponema pallidum* subspecies *pertenue*. The disease mainly affects the skin, but if untreated, can also affect bone, joints and cartilage. Yaws may persist for many years as a chronic infection, and late stage disease may lead to crippling disfigurement [1, 2]. The bacterium that causes yaws is closely related to *T*. *pallidum* ssp. *pallidum*, the causative organism of venereal syphilis, however *T*. *pallidum* ssp. *pertenue* is thought to be less virulent [3]. Transmission of yaws occurs from person to person through direct skin to skin contact, involving transfer of infectious exudates from the early skin lesions of infected individuals to micro- or macro-abrasions of the skin of siblings/playmates. [4–7].

Yaws was previously widespread throughout the tropics but a global eradication campaign between 1952 and 1964 resulted in a 95% reduction in disease prevalence worldwide [8]. Following this initial success, yaws control was integrated into national primary healthcare systems. Unfortunately, this integration resulted in a weakening of yaws surveillance in many countries and the re-emergence of the disease by the 1970s [9]. Several reports have recently documented a resurgence of yaws in parts of West and Central Africa, South East Asia and several Western Pacific Islands [10–12]. Yaws is an important public health problem in Ghana and affects the poor rural communities. The disease is reported in all the ten regions of the country. A prevalence study in three purposively selected districts in Southern Ghana in 2008 showed an overall prevalence of 1.92% among children in primary schools; however individual school prevalence rates ranged from 0% to a high of 19.5% [13].

Previous mass treatment campaigns to eradicate yaws in Ghana were based on the use of a single dose intramuscular (IM) injection of long-acting penicillin. The advantages of single-dose treatment with penicillin are its low cost, adherence and the absence of antimicrobial resistance despite extensive use. However, treatment with intramuscular penicillin has several disadvantages such as pain, anaphylaxis, and potential for transmission of other blood-borne infections.

Azithromycin has previously been shown to be an effective agent in the treatment of venereal syphilis [14]and is also the cornerstone of the strategy for the elimination of trachoma [15]. Azithromycin offers a number of advantages as an agent for the treatment of yaws, including its low cost, oral route of administration, excellent safety profile and negligible risk of anaphylaxis. As such, the drug is well suited for use in community mass treatment campaigns for yaws eradication [16]. In 2012, a single centre study conducted in Papua New Guinea, showed that a single oral dose of 30mg/kg azithromycin was non-inferior to a single injection of benzathine penicillin in the treatment of the disease, Clinical cure rate was 85.4% (95% CI: 78.2–90.6) in patients treated with azithromycin compared to 86.5% (95% CI: 79.5–91.5) in those treated with benzathine penicillin [17]. These findings led to recommendations incorporated into the WHO’s Morges yaws eradication strategy [18]. Here, we report a similar study carried out in Ghana, West Africa in order to confirm the efficacy and suitability of azithromycin for treatment of yaws in Sub-Saharan Africa.

## Methods

### Ethics Statement

This trial was conducted according to the principles of the Declaration of Helsinki. The study was approved by the Ghana Health Service Ethical Review Committee (Ref: GHS-ERC: 13/11/10). Written informed consent was obtained from parents/guardians of all participants; those aged 12 years and above also signed an assent certificate.

### Study Setting

**Fig 1:**
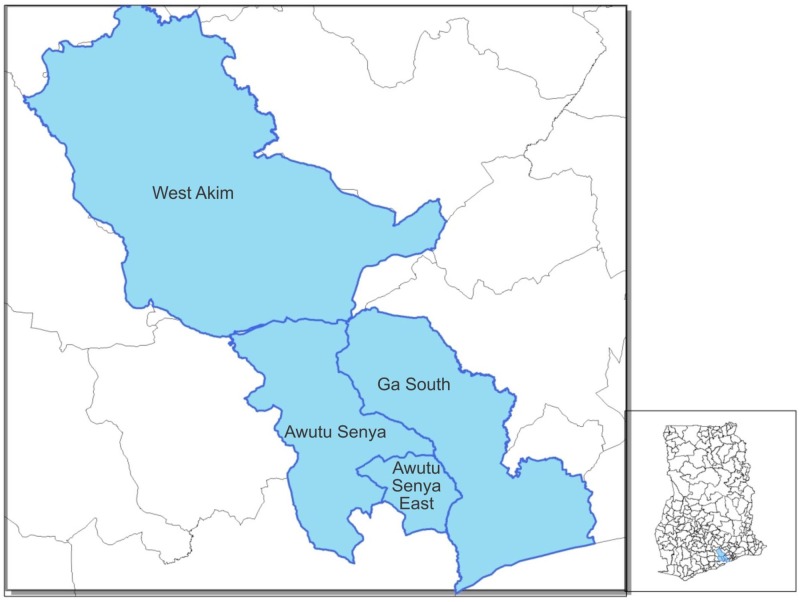
Study Districts Ga South, Awutu Senya and West Akim Districts of Ghana. In 2012 the Awutu Senya district was divided into Awutu Senya and Awutu Senya East districts.

The study was conducted between 25^th^ May 2011 and 31^st^ December 2012 in three neighbouring yaws endemic districts in southern Ghana: Ga South, Awutu Senya and West Akim districts (Fig 1). The three study districts have a total population of 787,747 with a distribution of 316,091, 274,584 and 197, 072 respectively. Children under the age of 15 years, known to bear the bulk of yaws infections, represent an estimated 38.3% of these populations. There are more than 600 rural communities in these three districts. The districts have a total of 8 government and private hospitals, 10 government health centres, 81 private health facilities made up of small clinics and maternity homes and 16 Community Based Health Planning and Services (CHPS) compounds. Only 20% of these health facilities are located in the rural parts of the district where yaws occurs. The doctor population ratio in the study area is 1:15,754.

The main occupation of the people in the study area is subsistence farming. There are a total of 1750 primary schools and kindergartens in the study area with a total enrollment of 174, 536 made up of 51% males and 49% females.

Yaws surveillance in the study area is mainly by passive detection at the health facilities. Based on routine reports of presentation of clinical cases to health facilities and a small number of active case searches in schools, the yaws case notification rate has been estimated to be between 87–241 per 100,000 people. Between 2009 and 2011, 2674 clinical cases of yaws were reported in the 3 study districts combined.

### Study Participants

Participants were recruited based on clinical suspicion by health workers at home or in school, and all subjects with suspected yaws were assessed for eligibility. Clinical examination involved inspection of the skin and scalp for signs of early yaws lesions. A suspected case of primary yaws was defined as an ulcer with raised edges and a dirty crusty base or a papilloma that appeared as a firm yellowish skin lesion with a dark tip, on any part of the body. A suspected case of secondary yaws was defined as any of the following skin lesions: multiple ulcerative or papillomatous skin lesions; a palmar or plantar hyperkeratosis or a macular, papular or maculopapular skin lesion. A confirmed case of yaws was defined as a suspected case with a positive TPHA test and an RPR titre of at least 1:4.

Photographs of the lesions were taken before treatment and subsequently at follow-up review visits. A 5mL sample of venous blood was collected from those with lesions clinically suggestive of yaws and analyzed at the National Public Health Reference Laboratory in Accra by qualitative *Treponema pallidum* haemaglutination assay (TPHA) testing (Debens Diagnostics Ltd., Ipswich, UK.). In parallel, a second set of frozen sera were sent to the Komfo Anokye Teaching Hospital serology laboratory in Kumasi for qualitative and quantitative rapid plasma reagin (RPR) testing (Immutrep RPR test kit, Omega Diagnostics, Alva UK).

Inclusion criteria for enrolment were individuals aged 1–15 years with suspected primary or secondary yaws, a reactive TPHA test and a RPR titre of at least 1:4. Exclusion criteria were a negative qualitative TPHA test, a baseline RPR titre of less than 1:4, allergy to penicillin and/or a macrolide antibiotic, a medical condition that would impair drug absorption, recent ingestion of a broad spectrum antibiotic (30 days prior to the day of randomisation) and patients who were not willing to give informed consent or who would not be available for follow up visits.

### Study Drugs

In this study azithromycin manufactured by Pfizer in the strength of 250mg tablets was supplied by Gokals Laborex, Ghana. Benzathine penicillin, 1.2 million units per vial, manufactured by Troge Medicals, Hamburg was supplied by Ghana Health Service Central Medical Stores.

### Randomisation Process

Participants who met the inclusion criteria were randomized to receive treatment with either a single dose oral azithromycin administered at a dose of 30mg/kg (maximum of 2g) or a single dose of benzathine penicillin administered as an intramuscular injection of 1.2 million units for subjects 10-15years, and 0.6 million units for those below 10 years of age. Participants in both arms were directly observed receiving treatment and for two hours thereafter.

The allocation sequence was based on a computer generated block randomization scheme, stratified according to district. Participants were allocated in a ratio of 1:1 to treatment with azithromycin or penicillin. Treatment allocation was concealed from investigators through the use of sequentially numbered, opaque, sealed envelopes that were kept in a safe and were opened at the point of treatment by the treatment team.

Owing to the obvious differences between the mode of administration of the two drugs, investigators and study participants could not be blinded to the treatment received, however individuals assessing study outcomes were masked to treatment allocation.

Serological tests were conducted in separate laboratories by laboratory technicians masked to clinical data.

### Follow—Up

Participants were followed up at 3 weeks, 3 months and 6 months. Skin lesions were re-examined at 3 weeks post treatment. At 3 months and 6 months, skin lesions were examined and blood collected for repeat quantitative RPR testing. Individuals with lesions that had not healed at follow-up were re-treated with benzathine penicillin. Health workers and community-based surveillance volunteers monitored and documented all adverse events up to 72 hours after treatment. Parents and teachers also were counselled on possible adverse events after the field team had departed and the need to report to the nearest health facility if necessary. Adverse events that occurred within 2 hours of treatment were documented and managed by the field teams.

Our primary outcome was clinical cure defined as a total or partial resolution of yaws skin lesions 3 weeks after treatment. The secondary outcome was serological cure defined as at least a 4-fold drop in baseline RPR titre within 6 months of treatment. Treatment was considered to have failed if there was no resolution of yaws skin lesions (complete or partial) 3 weeks after treatment.

### Statistical Analysis

This trial was designed to assess if azithromycin was non-inferior to benzathine penicillin for the treatment of yaws. With an expected efficacy of penicillin of 95%, a type 1 error of 0.05, and a non-inferiority margin of 10% and assuming that 10% would be lost to follow-up, a sample size of 310 children (155 per arm) would give a statistical power of 90% to test the hypothesis. Analysis of the primary endpoint of clinical cure was estimated by the two-sided 95% confidence interval for the difference in cure rates between the penicillin and azithromycin groups. Secondary outcome analyses were done using similar methods. Subgroup analyses were performed with stratification by baseline RPR titre, household exposure to yaws and stage of clinical yaws. A two-sided test at a significance level of 0.05 was used in the comparison of baseline characteristics of the two treatment groups. All statistical analyses for this study were carried out in STATA 11.1 (Statacorp, Texas, USA). The per-protocol (PP) analysis included all subjects who completed all study procedures at 6 months. The intention-to-treat (ITT) analysis included all eligible participants who were randomised and treated. Individuals with missing data were considered treatment failures for the purposes of the intention-to-treat analysis.

## Results

**Fig 2:**
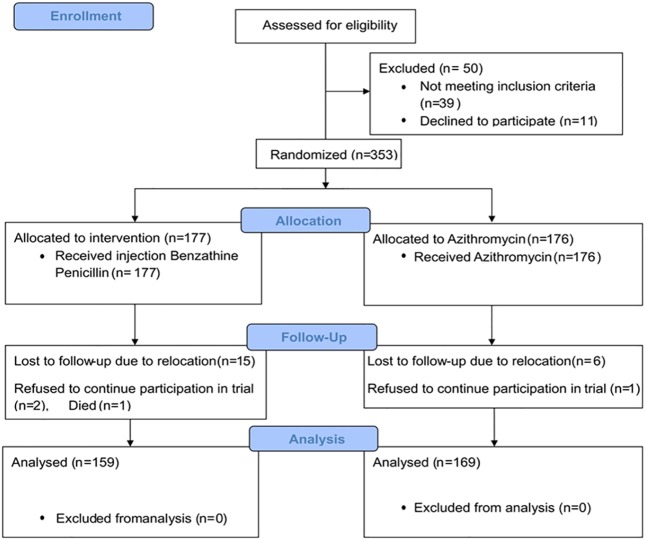
Trial Profile: Flow of patients through various stages of trial.

The trial profile is shown in Fig 2. From May 2011 to December 2012, four hundred and three subjects with suspected primary or secondary yaws lesions were assessed for eligibility; 50 were found to be ineligible (39 were either TPHA negative or had a RPR titre below 1:4, 11 declined to participate). Therefore 353 eligible participants were randomly assigned to receive either a single-dose oral azithromycin or a single intramuscular injection of long-acting penicillin. Of the 353 subjects randomised, 25 participants (7.0%) were lost to follow up. Six participants in the azithromycin group relocated and 1 refused to continue participation in the study. In the penicillin group, 15 participants relocated, 2 refused to continue participation and 1 patient died 5 months after treatment, a verbal autopsy concluded cause of death as malaria and National Yaws Eradication Programme was notified. The remaining 328 participants (169 in the azithromycin group and 159 in the penicillin group) completed the study and were analysed in the per protocol analysis.

Demographic and clinical characteristics did not vary between the two treatments groups (Table 1). The mean age of study participants across both groups was 9.5 years (SD: 3.1, range: 1 to 15 years); 274 (70%) were male. Primary yaws was present in 187 cases (53%), 13 participants (3.7%) had fever at presentation, 17 (4.8%) had arthralgia, and 33 (9.4%) had one or more other skin lesions in addition to those of yaws. One hundred and fifty five (43.9%) participants had a baseline RPR titre between 1:4 and 1:16, and 198 (56.1%) had titres between 1:32 and 1:128. One hundred and seventy one participants (48.6%) lived in houses with at least one other individual who had been diagnosed with active yaws within the past one month.

**Table 1:** Baseline Characteristics of Intention- to- Treat Population.

Characteristic	Total Enrolled n = 353	Benzathine Penicillin n = 177	Azithromycin n = 176
**Age**			
Mean age (years)	9.5 (SD:3.1)	9.7 (SD:3.1)	9.3(SD:3.1)
1–5 years	39(11%)	19(10.7%)	20(11.4%)
6–15 years	314(89%)	158(89.3%)	156(88.6%)
**Sex**			
Males	247(70%)	124(70%)	123(70%)
Females	106(30%)	53(30%)	53(30%)
**Baseline Clinical Presentation**			
Primary yaws	187(53%)	94(53%)	93(52.8%)
Secondary yaws	166(47%)	83(47%)	83(47.2%)
Arthralgia	17(4.8%)	6(3.4%)	11(6.3%)
Other Skin Lesions	33(9.4%)	15(8.5%)	18(10.2%)
Fever	13(3.7%)	4(2.3%)	9(5.1%)
**Baseline RPR titre**			
1:4 to 1:16	155(43.9%)	76(42.9%)	79(44.9%)
1:32 to 1:128	198(56.1%)	101(57.1%)	97(55.1%)
*Household Exposure to yaws	171(48.6%)	84(47.7%)	87(49.4%)

RPR:Rapid Plasma Reagin.

* Children living in same house as other children with suspected yaws lesions

The most frequent clinical lesions were ulcers (167, 47.3%) followed by papillomas (101, 28.6%), hyperkeratosis of the palms and soles (25, 7.1%), while the rest were macules, papules and maculopapular lesions. Three individuals had ulcers with sabre tibia.

**Fig 3:**
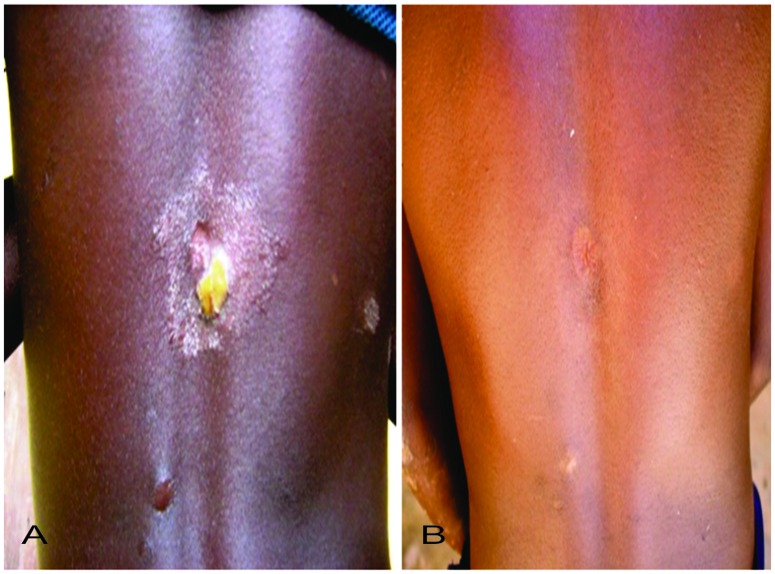
Before and After Treatment with Azithromycin Papillomatous ulcer at the back of patient showing complete healing after 3 weeks of treatment with azithromycin: A. Before Treatment, B. After Treatment.

**Fig 4:**
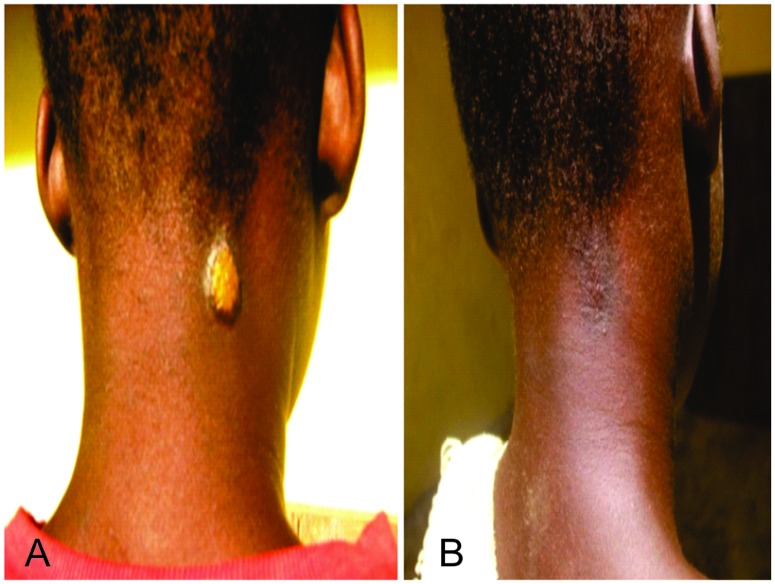
Before and After Treatment with Benzathine penicillin Papillomatous ulcer on the neck of patient showing complete healing after 3 weeks of treatment with benzathine penicillin: A. Before Treatment, B. After Treatment.

Similar cure rates were recorded between the two treatment groups (Table 2). For the primary outcome of clinical cure defined as complete or partial healing of yaws lesion, 166 out of 169 participants (98.2%) in the azithromycin group and 155 out of 159 participants (96.9%) in the penicillin group exhibited complete or partial resolution (Figs 3 and 4)) 3 weeks after treatment (risk difference: -1.3%, (-4.7 to 2.0). For the secondary outcome (serological cure) 97 out of 169 (57.4%) participants in the azithromycin group showed a 4- fold or greater decline in baseline RPR titres by 6 months after treatment compared to 78 out of 159 participants (49.1%) in the penicillin group,(risk difference:-8.3 (-19.1 to 2.4). Azithromycin therefore met the criteria for non-inferiority in both the primary and secondary outcomes.

**Table 2:** Treatment Outcomes (PP).

N = 328	Benzathine penicillin (95% CI)	Azithromycin (95% CI)	Risk difference % (95%CI)
**Primary Outcome:**			
Clinical cure at 3 weeks	96.9%(94.1–99.6)	98.2%(96.2–100)	-1.3(-4.7 to 2.0)
**Secondary Outcome:**			
Serological cure at 6 month	49.1% (41.2–56.9)	57.4% (49.9–64.9)	-8.3 (-19.1 to 2.4)

Three (1.8%) participants in the azithromycin group and 4 (2.5%) in the penicillin group with ulcerative yaws lesions did not resolve 3 weeks after treatment and were classified clinically as “treatment failures”. All participants considered as “treatment failures” were re-treated with intramuscular penicillin and all ulcers subsequently resolved within 2 weeks. Five subjects with ulcerative lesions which had healed at 3 weeks and had achieved serological cure were found to have recurred during the 3 month follow up period. Of these 3 (1.7%) occurred in the azithromycin group and 2 (1.3%) in the penicillin group. However due to the fact that subjects had achieved serological cure, lesions were likely of a different aetiology.

Azithromycin treatment also proved to be non-inferior to penicillin therapy in subgroup analyses of primary outcome (clinical cure at 3 weeks) by clinical stage of yaws, baseline RPR titre and household exposure (Table 3).

**Table 3:** Sub Group Analysis of Primary Outcome (Clinical Cure at 3 Weeks).

	Benzathine penicillin (95% CI)	Azithromycin (95% CI)	Risk difference % (95%CI)
**Clinical Stage of yaws:**			
Primary	98.7(96.2–100)	98.9(96.7–100)	-0.2(-3.4 to 3.1)
Secondary	95.0(90.1–99.9)	97.5(93.9–100)	-2.5(-8.4 to 3.4)
**Baseline RPR titre:**			
1:4–1:16	93.7(87.5–99.7)	98.6(96.0–100)	-5.0(-11.6 to 1.6)
1:32–1:128	99.0(96.7–100)	97.9(95.0–100)	1.1(-12.5 to 4.6)
**Household Exposure:**			
Exposed	98.7(96.1–100)	92.6(94.2–100)	1.1(-3.4 to 5.3)
Not exposed	95.1(90.2–99.9)	98.8(96.5–100)	-3.8(-9.0 to1.5)

Analysis of serological cure by baseline RPR titres are shown in Table 4. Cure rates were low (41.8% in the azithromycin group and 31.7% in the penicillin group) in patients with low RPR titres of 1:4–1:16 compared to higher cure rates in patients with high RPR titres of 1:32–1:128 (69.5% in the azithromycin group and 60.4% in the penicillin group).

**Table 4:** Analysis of Serological Cure by Baseline RPR Titre.

Baseline RPR Titre	Benzathine penicillin (95% CI)	Azithromycin (95% CI)	Risk difference % (95%CI)
1:4–1:16	31.7(21.2–44.5)	41.8(31.0–53.6)	-10.1(-26.0to 5.9)
1:32–1:128	60.4(50.1–69.8)	69.5(59.3–78.0)	-9.1(-22.0 to 4.4)

No serious adverse effects related to the treatment drugs were reported in this trial. Minor adverse effects were reported by 4 participants (2.4%) in the azithromycin group, most commonly gastrointestinal upset, and 8 participants (5%) in the penicillin group, most commonly pain at the injection site.

## Discussion

This study shows that a single oral dose of azithromycin given at a dosage of 30mg/kg is non-inferior to a single intramuscular dose of benzathine penicillin in the treatment of yaws in children in Ghana. Indeed the cure rate for the primary outcome at 3 weeks was slightly in favour of azithromycin. For the primary outcome of clinical cure, 166 out of 169 participants (98.2%) in the azithromycin group and 155 out of 159 participants (96.9%) in the penicillin group showed a complete or partial resolution of yaws lesions 3 weeks after treatment. Our results were consistent with a previous study conducted in Papua New Guinea (17). We also showed that azithromycin was non-inferior to IM penicillin in all subgroup analyses, confirming the robustness of this conclusion.

Azithromycin was well tolerated by participants, with no serious adverse events reported after treatment. The recorded side effects were, in keeping with the known side effects profile of the drug, namely mild to moderate and most commonly gastrointestinal in nature.

Serological cure rates at 6 months were higher in the former study in Papua New Guinea (88%) compared to this study (57.4%), due to enrolment of patients with higher baseline RPR titres (≥1:16) in the PNG study. In this trial, 42.6% of participants treated with azithromycin and 50.9% of those treated with penicillin who were clinically cured did not achieve a decline in baseline RPR titres by at least 2 dilutions (4-fold) by 6 months after treatment. Among patients treated with azithromycin 5.3% did not achieve a fall in RPR titre, 38.1% achieved a 2 fold fall, 2.9% showed a 2 fold increase and 2.4% had a 4 fold increase in RPR titre 6 months after treatment. In the penicillin group 12.7% did not show a fall in RPR titre, 33.5% showed a 2-fold falling titre, 11.8% showed a 2 fold increase in titre and 0.6% showed a 4 fold increase in titre 6 months after treatment.

Unfortunately, it is clinically and serologically impossible to distinguish treatment failure and relapse from re-infection. Since this study was conducted in endemic communities where exposure to antibiotics is uncommon, it is possible that many of those cases that showed a 4-fold increase in titre reflect re-infection rather than treatment failure. Increase in RPR titre could also be related to performing of initial RPR titre in the early stage in infection before titre reached its peak. The 2-fold increases and decreases in titre that were recorded here largely reflect the relative insensitivity of the quantitative RPR test.

The availability of an orally effective treatment for yaws is key if the goal of eradication of yaws is to be attained. In this trial we demonstrate the efficacy of azithromycin in the treatment of yaws in a second yaws-endemic region far removed from the initial trial site in Papua New Guinea, confirming its place in the WHO yaws eradication strategy [19]. Effective treatment of yaws involves treatment of whole communities. Azithromycin is well suited to administration by community health volunteers, even in the poorly-resourced rural communities where yaws occurs. A recent study has demonstrated the impact of a single round of mass treatment with azithromycin in reducing transmission of yaws in Lihir Island, Papua New Guinea [20].

Azithromycin treatment failure among patients with syphilis, caused by a closely-related treponeme *T*. *pallidum* ssp. *pallidum*, has been widely reported in high-resource settings where overuse of antibiotics is common. Treatment failure has been associated with a single amino acid mutation at positions 2058 and 2059 in the 23S rRNAgene [21], which prevents binding with the bacterial 50S ribosomal subunit. In contrast, azithromycin resistance among *T*. *pallidum* ssp. *pertenue* strains has yet to be documented.

Although populations in yaws endemic areas are typically not exposed to excessive antibiotic usage, there is a clear need to strengthen surveillance systems and closely investigate possible treatment failures for evidence of emergence of azithromycin resistance.

There are a number of limitations to this study. Most notable is the inability to mask treatment assignments. We tried to mitigate this by having outcomes evaluated by independent assessors who were blinded to treatment allocation. We could not determine whether lesions that did not heal at 3 weeks post treatment were true treatment failures, since neither dark field microscopy nor PCR of lesion exudates was performed. As mentioned above, we were also unable to distinguish treatment failure from re-infection. Enrolment of patients with low RPR titres (1:4–1:8) may have influenced serological cure in this study, it was impossible to observe a 4-fold drop in RPR in patients with low titres. Finally, participants were followed up for only 6 months, after which no serological or clinical data were collected.

This randomized controlled trial has clearly demonstrated that a single oral 30mg/kg dose of azithromycin is non inferior to a single dose of IM benzathine penicillin for the treatment of early yaws in Ghana. There was no significant difference in cure rates between patients treated with azithromycin and those treated with injection benzathine penicillin. Oral treatment with azithromycin overcomes the logistical and operational problems of using intramuscular penicillin in mass treatment campaigns. Our findings lend additional support for the use of a single dose azithromycin as the preferred regimen in yaws eradication programs.

## Supporting Information

S1 Text STUDY PROTOCOL. (PDF)

S2 Text ETHICAL CLEARANCE. (PDF)

S3 Text CONSORT CHECKLIST. (PDF)

S1 Table STUDY DATA. (XLSX)
