# Supplementary figures and images for: A Single Dose Oral Azithromycin versus Intramuscular Benzathine Penicillin for the Treatment of Yaws-A Randomized Non Inferiority Trial in Ghana
Source: PLoS Negl Trop Dis. 2017 Jan 10;11(1):e0005154. doi: 10.1371/journal.pntd.0005154 (PMC5224786; doi:10.1371/journal.pntd.0005154)

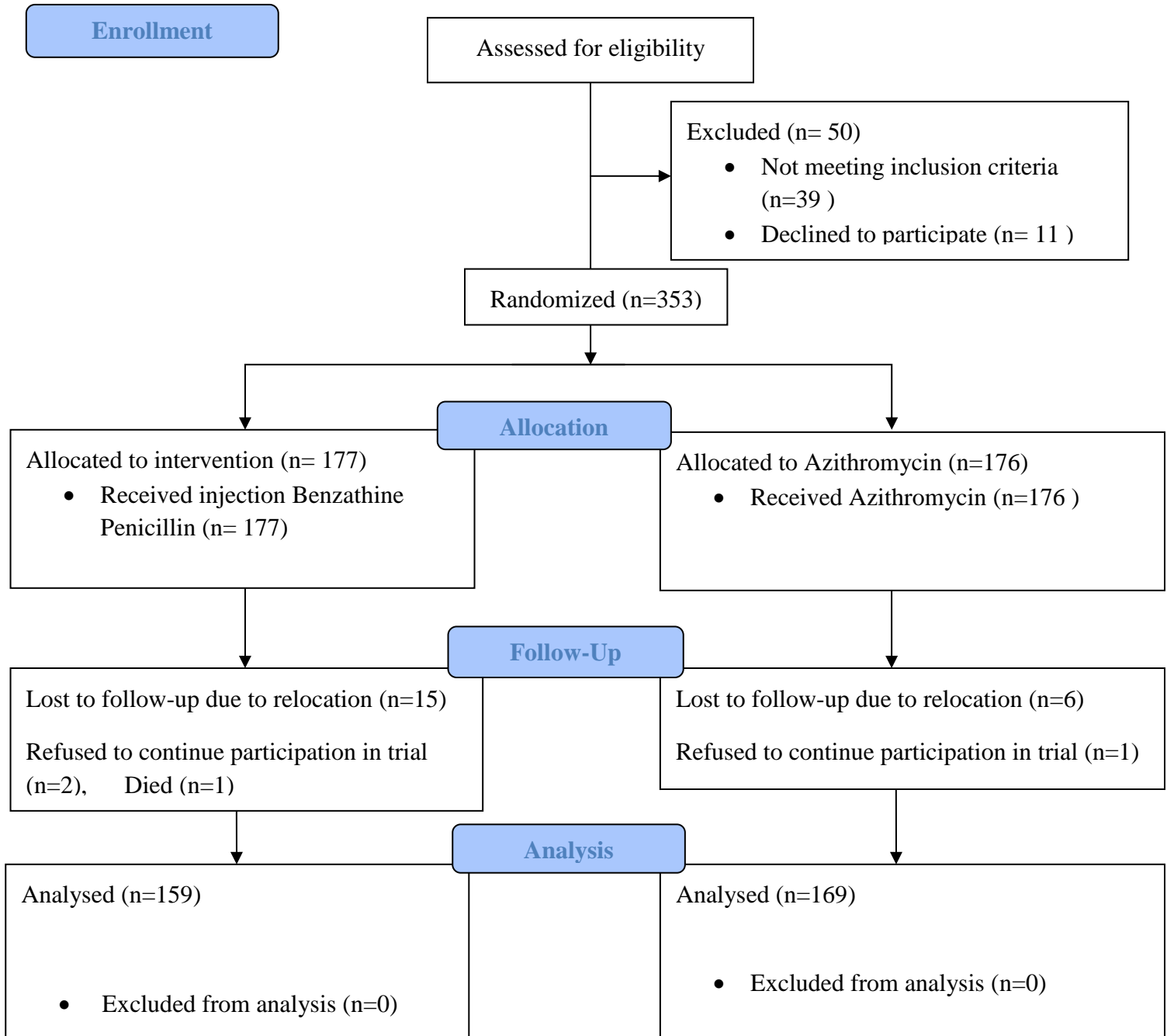

Supplement: S1 Text — (PDF) [file pntd.0005154.s001.pdf]
