# Supplementary material for: A Single Dose Oral Azithromycin versus Intramuscular Benzathine Penicillin for the Treatment of Yaws-A Randomized Non Inferiority Trial in Ghana
Source: PLoS Negl Trop Dis. 2017 Jan 10;11(1):e0005154. doi: 10.1371/journal.pntd.0005154 (PMC5224786; doi:10.1371/journal.pntd.0005154)
Supplement: S2 Text — (PDF) [file pntd.0005154.s002.pdf]

APPENDIX J

GHANA HEALTH SERVICE ETHICAL REVIEW COMMITTEE

In case of reply the number and date of this *<u>Letter should be quoted.</u>*

My Ref. :GHS-ERC: 3
Your Ref. No.

Research & Development Division
Ghana Health Service
P. O. Box MB 190
Accra

Tel: +233-0302-681109
Fax + 233-0302-685424
Email: Hannah Frimpong@ghsmail.org

February 25 , 2011

DR. CYNTHIA KWAKYE-MACLEAN, Principal Investigator

ETHICAL CLEARANCE - ID NO: GHS-ERC: 13/11/10

The Ghana Health Service Ethics Review Committee has reviewed and given approval for the implementation of your Study Protocol titled:

"Oral Azithomycin in the treatment of Yaws – A randomized clinical trial in the Ga West Municipality"

This approval requires that you submit periodic review of the protocol to the Committee and a final full review to the Ethical Review Committee (ERC) on completion of the study. The ERC may observe or cause to be observed procedures and records of the study during and after implementation.

Please note that any modification of the project must be submitted to the ERC for review and approval before its implementation.

You are also required to report all serious adverse events related to this study to the ERC within seven days verbally and fourteen days in writing.

You are requested to submit a final report on the study to assure the ERC that the project was implemented as per approved protocol. You are also to inform the ERC and your mother organization before any publication of the research findings.

Please always quote the protocol identification number in all future correspondence in relation to this protocol

SIGNED.......................................
DR. CYNTHIA BANNERMAN
(GHS-ERC VICE CHAIRMAN)

Cc: The Director, Research & Development Division, Ghana Health Service, Accra
